# Exercises in Equine Surgery

**Published:** 1897-09

**Authors:** 


EXERCISES IN EQUINE SURGERY. By P. J. CADIOT, Professor of the Alfort Veterinary School. Translated by A. W. BITTING, D.V.M., Veterinarian to Purdue University and Agricultural Experiment Station. Edited by Prof. A. LIAUTARD, M.D., V.M., Dean of the American Veterinary College. Published by William R. Jenkins, No. 853 Sixth Avenue, New York City.

WE wish that such an act would become a fixed custom in our country as the one performed by Dr. A. W. Bitting in presenting as a thesis at the close of his college career this worthy translation of *Exercises in Equine Surgery*. The veterinary profession and English veterinary literature would be so greatly enriched by such additions as this as soon to lead to more of our own very able men becoming writers. This work, so well known and highly appreciated among the students and busy practitioners of France and many parts of Europe, is so complete and so valuable in the field it covers that no American veterinary surgeon will want to take up any of the many equine surgical operations without reviewing for a moment the ever-thoughtful suggestions and reminders contained in this book. Fully illustrated, and added to by the editor, Prof. A. Liautard, it comes to us in a thoroughly acceptable style and attractive form, made so by that long-experienced veterinary publishing house of W. R. Jenkins, of New York, that has produced from its presses so many valuable contributions to veterinary literature. We are sure this will prove one of the most sought-for books ever issued by this house.

---

*The Veterinary Blue-Book* of New York County and of veterinarians in the United States is now announced for publication in October next. Any changes of addresses or positions are asked for by the Chairman of the Publication Committee, Dr. Rush S. Huidekoper, who may be addressed until September 30, 1897, at Weaver Avenue, Newport, Rhode Island.

---

The Chicago Live-stock Exchange annually expend $1800 to inspect and control actinomycotic cattle coming into their yards.

